# New targets for old diseases: lessons from mucolipidosis type II

**DOI:** 10.1002/emmm.201303496

**Published:** 2013-11-08

**Authors:** Carmine Settembre, Andrea Ballabio

**Affiliations:** 1 Telethon Institute of Genetics and Medicine (TIGEM)Naples, Italy; 2 Department of Molecular and Human Genetics, Baylor College of MedicineHouston, TX, USA; 3 Jan and Dan Duncan Neurological Research Institute, Texas Children HospitalHouston, TX, USA; 4 Department of Translational and Medical Sciences, Medical Genetics, Federico II UniversityNaples, Italy

**Keywords:** lysosomal storage disorders, lysosome, osteoblast, osteoclast, skeleton

Lysosomal storage disorders (LSDs) are inherited diseases characterized by progressive intracellular accumulation of undigested macromolecules within the cell due to specific lysosomal defects. Lysosomal storage results in a global impairment of many lysosome-dependent pathways (*e.g*. autophagy and endocytosis), leading to cellular dysfunction and death (Ballabio & Gieselmann, 2009). LSD patients often have a complex phenotype, with visceral, skeletal, haematological and neurological involvement. Several mechanisms underlie the etiopathogenesis of the LSDs and their relative contribution may vary, depending on the tissue and cell type. For instance, a generalized inflammatory process affecting multiple tissues has been observed in several mouse models of LSDs, although its role in disease pathogenesis remains still largely unclear (Ballabio & Gieselmann, 2009).

Over 50 different LSDs have been described to date, which are due to mutations in genes that encode soluble hydrolases, membrane proteins and lysosomal accessory proteins, resulting in the block of a specific catabolic pathway. Among the lysosomal accessory proteins, one notable example is that of the α and β subunits of the *N*-acetylglucosamine phosphotransferase complex, which are encoded by the *GNPTAB* gene. This complex catalyses the post-translational incorporation of a mannose 6-phosphate (M6P) residue on newly synthesized lysosomal enzymes (Bao et al, 1996). This glycosylation event is crucial for the correct M6P receptor-dependent targeting of lysosomal enzymes to lysosomes (Braulke & Bonifacino, 2009). In patients affected by mucolipidosis type II (MLII), a LSD due to mutations of the *GNPTAB* gene, lysosomal enzymes are missorted to the extracellular space. This results in intra-lysosomal deficiency of multiple enzymes and consequent accumulation of undigested substrates in several organs and tissues, particularly the skeletal system. The pathological mechanisms leading from cellular storage defects to skeletal abnormalities in MLII patients (Schweizer et al, 2013) are poorly understood; it is therefore crucial to improve our understanding in this respect to help develop possible therapeutic strategies.

Skeletal development and homeostasis are coordinated processes that are controlled by three main cell types: chondrocytes in cartilage and both osteoblasts and osteoclasts in bone (Karsenty et al, 2009). During puberty, longitudinal bone growth is mainly regulated by chondrocyte proliferation, differentiation and matrix deposition, whereas matrix calcification (hence bone formation) is controlled by the opposed and finely balanced activities of osteoblasts, the bone forming cells and osteoclasts, the bone resorbing cells. Deregulation of this balance leads to many severe bone disorders, such as osteopetrosis and osteoporosis. It is thus not surprising that several regulatory signalling molecules and intracellular pathways co-regulate the activity of osteoclasts and osteoblasts to maintain bone homeostasis (Karsenty et al, 2009).

Osteoclast activity is thought to be highly dependent on lysosomal function through the generation of the resorption lacunae or pits, sealed extracellular low-pH environments enriched in lysosomal proteases, where bone matrix degradation occurs (Lacombe et al, 2013). Indeed multiple forms of human osteopetrosis are caused by mutations in genes encoding for lysosomal proteins. Of note, the fine cellular regulation of lysosomal exocytosis is an exciting area of research, which only recently has begun to develop (DeSelm et al, 2011; Ferron et al, 2013; Medina et al, 2011).

In this issue of *EMBO Molecular Medicine*, Kollmann et al (2013) report on their studies of the molecular mechanisms underlying the skeletal phenotype observed in MLII. To this end, they designed an MLII knock-in mouse model that carries the insertion of a cytosine in the murine *Gnptab* gene (c.3082insC), a specific mutation observed in an MLII patient. This insertion interrupts the Gnptab open reading frame leading to a truncated GlcNAc-1-phosphotransferase protein that is not cleaved to the mature α-subunit and thus cannot exit the endoplasmic reticulum. The skeletal features of MLII mice closely mimic those observed in human MLII patients and are characterized by growth retardation and multiple skeletal dysostosis.

**Figure 1:**
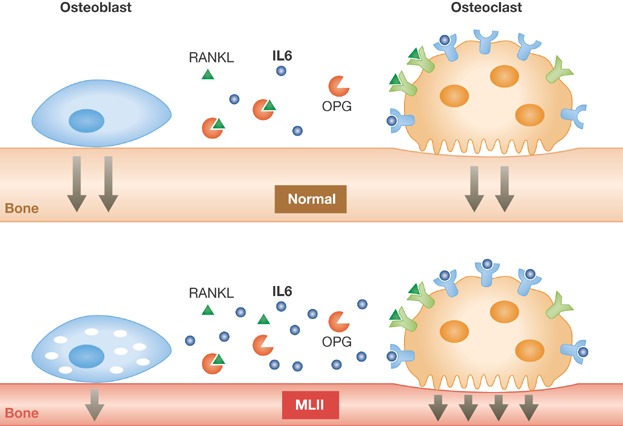
In normal conditions, the activities of osteoblasts and osteoclasts are co-regulated by locally produced factors such as RANKL, osteoprotegerin (OPG) and IL6 In MLII mice lysosomal dysfunction in osteoblasts triggers the secretion of higher levels of IL6. Since IL6 is a cytokine with a potent pro-osteoclastogenic activity, as a result MLII patients have increased osteoclast, and thus bone resorptive activity.

The authors observed severe reduction of trabecular bone volume associated with an increased cortical porosity in the MLII mice compared to control mice. Interestingly, this phenotype appeared to be the consequence of an unbalanced ratio between osteoblast and osteoclast activities, whereby bone formation was severely reduced and bone resorption strongly increased. To gain molecular insight, the authors isolated and cultured primary osteoblasts and osteoclasts from control and MLII mice. Primary osteoblasts isolated from MLII mice showed impaired differentiation capacity *in vitro* that was associated with reduced mineralization. Conversely, cultured osteoclasts from MLII mice showed normal differentiation and resorptive capacities compared to control cells. Furthermore, the cytosol of osteoblasts appeared to be vacuolized reflecting lysosomal storage, while the cytosol of osteoclasts did not show obvious differences compared to control cells. The data thus indicate that osteoclasts in the MLII knock-in mouse are not affected by the lysosomal enzyme missorting and that the increase in their activity and number *in vivo* may be the result of a non-cell autonomous effect (Fig 1).

… lysosomal dysfunction in a given cell type can contribute to the pathological phenotype not only by perturbing function in the affected cells, but also by altering crosstalk with other cells.

Osteoclasts are large multinucleated cells that originate from the monocyte–macrophage cell lineage and whose differentiation is controlled by several cytokines produced by surrounding cells, such as osteoblasts. To explore the possibility that increased osteoclastogenesis in MLII mice was the consequence of altered signals produced by surrounding cells, the authors analysed the transcriptome of MLII osteoblasts and found an increased expression of IL6, a cytokine known to have potent pro-osteoclastogenic activity. Interestingly, the increased IL6 expression levels appeared to be the direct consequence of lysosomal storage since this phenomenon was also observed in other cell types isolated from the MLII mice. The data strongly suggest that this family of cytokines may play a broader role in the pathogenesis of MLII. In this respect, it is worth noting that in a different model of LSD with severe lysosomal storage, mucopolysaccharidosis type VII, the levels of IL6 family members were found decreased in the growth plate of affected mice (Metcalf et al, 2009). Further studies will be required to understand whether this class of cytokines might indeed be a novel therapeutic target for the treatment of LSDs. A message emerging from this elegant study, as well as from previous observations (Simonaro et al, 2010), is that the immune system plays a deleterious role in skeletal pathogenesis pathogenesis of LSDs. Indeed, on going preclinical trials using anti-inflammatory drugs to treat the skeletal phenotype of the mouse model of mucopolysaccharidosis type VI, are yielding promising results (Eliyahu et al, 2011). Another important message from the paper by Kollmann et al (2013) is that lysosomal dysfunction in a given cell type can contribute to the pathological phenotype not only by perturbing function in the affected cells, but also by altering crosstalk with other cells. A similar mechanism was recently suggested to explain some of the neurological features observed in a different model of LSD (multiple sulfatase deficiency), in which lysosomal storage in astrocytes was sufficient to trigger neuronal dysfunction, hence providing yet another example of a non-cell autonomous pathomechanism in LSDs (Di Malta et al, 2012).

Given the fact that osteoclast hyperactivity in the MLII mouse model appeared to be the consequence of altered signalling rather than of a cell autonomous defect, the authors also attempted to pharmacologically modulate osteoclast activity. To this end, control and MLII mice were treated with alandronate, a well-known, potent anti-resorptive bisphosphonate, which inhibits osteoclast function. Compared to the placebo-treated controls, MLII mice treated with alandronate showed normalized bone parameters and rescued bone mass defects. These important results demonstrate that a major pathological feature of MLII is indeed potentially treatable using an FDA approved drug currently in therapy for the treatment of several bone diseases. Increased bone resorption parameters were also present in bone biopsies isolated from an MLII patient, strongly suggesting that the same therapeutic strategy may be applied to human patients.

… a major pathological feature of MLII is indeed potentially treatable using an FDA approved drug currently in therapy for the treatment of several bone diseases.

This important study exemplifies how the convergence of cell biology and mouse genetics may significantly accelerate the identification and design of new strategies for the treatment of LSDs, in particular for those organs that are especially difficult to target with conventional therapies, such as bone and brain. Further studies are needed to explore whether the aberrant crosstalk between different organs plays an even broader role in the multisystemic phenotype of LSDs.

The authors declare that they have no conflict of interest.
